# Prevalence and factors associated with sputum smear non-conversion after two months of tuberculosis treatment among smear-positive pulmonary tuberculosis patients in Rwanda: a cross-sectional study

**DOI:** 10.1186/s12879-023-08395-6

**Published:** 2023-06-15

**Authors:** Gaetan Gatete, Kato J Njunwa, Patrick Migambi, Joseph Ntaganira, Albert Ndagijimana

**Affiliations:** 1 Department of Epidemiology and Biostatistics, School of Public Health, College of Medicine and Health Sciences, University of Rwanda, Kigali, Rwanda; 2 Rwanda Biomedical Center, Kigali, Rwanda; 3 School of Health Sciences, College of Medicine and Health Sciences, University of Rwanda, Kigali, Rwanda

**Keywords:** Tuberculosis, Factors, Sputum non-conversion, Rwanda

## Abstract

**Background:**

Non-conversion of sputum smear prolongs the infectivity of pulmonary tuberculosis patients and has been associated with unfavorable tuberculosis (TB) treatment outcomes. Nevertheless, there is a limited evidence on predictors of sputum smear non-conversion among smear-positive PTB (SPPTB) patients in Rwanda. Therefore, this study aimed to determine the factors associated with sputum smear non-conversion after two months of treatment among SPPTB patients in Rwanda.

**Methods:**

A cross-sectional study was conducted among SPPTB patients registered in the national electronic TB reporting system by all health facilities countrywide (Rwanda) from July 2019 to June 2021. Eligible patients who had completed the first two months of anti-TB treatment and with smear results at the end of the second month of treatment were included in the study. Bivariate and multivariate logistic regression analyses were done using STATA version 16 to determine the factors associated with sputum smear non-conversion. Adjusted odds ratio (OR), 95% confidence interval (CI), and p-value < 0.05 was considered statistically significant.

**Results:**

This study included 7,211 patients. Of them, 632 (9%) patients had sputum smear non-conversion at the end of the second month of treatment. In multivariate logistic regression analysis, age groups of 20–39 years (AOR = 1.7, 95% CI: 1.0-2.8) and 40–59 years (AOR:2, 95% CI: 1.1–3.3), history of first-line TB treatment failure (AOR = 2, 95% CI: 1.1–3.6), follow-up by community health workers(CHWs) (AOR = 1.2, 95% CI: 1.0-1.5), BMI < 18.5 at TB treatment initiation (AOR = 1.5, 95% CI: 1.2–1.8), and living in Northern Province of Rwanda (AOR = 1.4, 95% CI: 1.0–2.0), were found to be significantly associated with sputum smear non-conversion after two months of treatment.

**Conclusion:**

Sputum smear non-conversion among SPPTB patients remains low in Rwanda compared to countries of similar health care setting. Identified risk factors for sputum smear non-conversion among SPPTB patients in Rwanda were age (20–39 years, 40–59 years), history of first-line TB treatment failure, follow up by CHWs, BMI < 18.5 at TB treatment initiation and residence (Northern province).

## Background

Tuberculosis (TB) disease is due to an infection from *Mycobacterium tuberculosis* (MTB) transmitted through the airborne route, and it was the leading killer infectious diseases before the COVID-19 pandemic [1, 2]. An estimate of around one-third of the world’s population is infected with MTB [3]. Among these, in 2019, 10 million people had developed TB, 1.4 million had died from TB, and 95% of TB cases and 98% of TB deaths had occurred in developing countries [4, 5]. The majority of TB cases occurred in Asia and Africa at (44%) and (26%) respectively [6]. In Rwanda, TB is among the leading health problems with an incidence rate of 58 per 100,000 population; HIV-negative TB mortality rate of 2.5 per 100,000 population, and HIV-positive TB mortality rate of 2.5 per 100,000 population were reported in 2020 [7]. In the same year, TB associated deaths were 6% and 14.8% in bacteriologically diagnosed cases and clinically confirmed cases respectively [8].

Sputum smear conversion after two months of TB treatment is considered as a useful indicator in monitoring the performance of TB control programs and it is an important predictor for bacteriological cures [9, 10]. Non-conversion of sputum smear at the end of the intensive phase of treatment (first two months of TB treatment) is the main contributor to unfavorable TB treatment outcome [11, 12]. Studies conducted in different settings have identified numerous factors associated with sputum smear non-conversion among pulmonary TB (PTB) patients like diabetes, high bacillary load at baseline and advanced age [13–15]. In Rwanda, PTB patients treated in health facilities from rural area were found to be more likely to have low sputum conversion rate compared to those treated in health facilities from urban area [5].

In Rwanda, Drug-susceptible TB patients are treated with the standard first-line regimen of a two months phase (intensive phase) of rifampicin, isoniazid, pyrazinamide and ethambutol (2RHZE) followed by a four months phase (continuation phase) of rifampicin and isoniazid (4RH) [16]. Bacteriological follow-up of first-line treatment is done by a sputum smear examination which is performed at the end of the 2nd, 5th and 6th months of treatment, non-converters at two months continue a four treatment course and encouraged to adhere to treatment and monitored rigorously by CHWs or clinicians depending on their means of follow up and will be tested again at the fifth month of treatment [17].

TB surveillance system in Rwanda start at the community level where by CHWs identify people with TB symptoms (cough of more than 2 weeks, weight loss and night sweating) and refer them to the nearest health facility for diagnosis. At health facility, TB is detected through different entry points like out-patient department, hospitalization, HIV clinic, and other departments. In Rwanda TB is diagnosed using microcopy and GeneXpert as well as X-ray modalities. After TB diagnosis patients are enrolled in DOT services where the patients choose by his/her own to be followed at the health facility or in the community by CHWs.

Despite a good progress in fighting against TB, the World Health Organization (WHO) had highlighted that even in a good performing national TB program, there is a proportion of PTB patients who may still be smear-positive at the end of the intensive phase, regardless of a good adherence and directed medication [5]. In Rwanda, 20% of PTB patients were reported in 2007 as having sputum smear non-conversion after intensive phase [18]. However, few studies have been carried out to identify factors associated with this non-conversion after intensive phase in Rwanda. This study aimed to determine the prevalence and factors associated with sputum smear non-conversion after two months of TB treatment among SPPTB patients in Rwanda.

## Methods

### Study design and setting

The study was a cross-sectional study and consisted in reviewing abstracted retrospective records (data) of SPPTB patients registered at TB clinics in all health facilities in Rwanda using the national electronic TB reporting system (e-TB) from July 2019 to June 2021. This period was chosen because July 2019 is when the TB cases reported by all health facilities both urban and rural health facilities across the country shifted to reporting individual data in the e-TB. Since 2012, the Ministry of Health in Rwanda has been reporting health data in DHIS2. DHIS2 was customized to collect aggregate data of different areas of program interventions. This has been the first step moving forward, customizing DHIS2 to support data collection of aggregated data from all health facilities.

Alongside the quarterly report of aggregated data, the second step was to transform the paper-based register in a web-based electronic register with individual data. This electronic register has two objectives: to improve data quality and to improve quality of case management. The latter involves a reminders system (SMS) towards TB patients, to remind, among others, treatment compliance (avoid lost to follow up patients, tracking transferred patients, decrease in number of doses not taken on time) and better identification of patients requiring special management, such as HIV-positive TB patients, eligibility to special lab techniques.

Since August 2018, the National TB Program decided to only collect individual data of TB cases to make e-TB functional and usable in some health facilities, and to reduce the workload to improve quality of data in e-TB. Improvements include: data entry form simplified to be user friendly, the number of data elements reduced, the creation of Program indicators, involvement of TB Focal persons to enter data rather than data managers.

From July 2019 to June 2021, a total of 548 TB clinics/health facilities reported TB cases in the e-TB reporting system countrywide. The majority of TB clinics in Rwanda are located in the Southern Province with 142 (25.9%), other provinces like the Western Province has 131 (23.9%), Eastern Province has 129 (23.5%), Northern Province with 99 (18.6%) and Kigali City with 47 (8.6%) TB clinics.

### Study population

This study was conducted among all SPPTB patients reported in e-TB system from July 2019 to June 2021. Were included in the study patients who had completed at least the intensive phase of first line of TB treatment, and whose follow-up sputum smear results after the second month of TB treatment were recorded. Smear-positive PTB patients without records of follow-up sputum smear examination results after the second month of TB treatment and patients who had died or had interrupted treatment before the end of the intensive phase were excluded from the study.

### Sampling

In this we used a take all sample approach, all SPPTB patients reported in the e-TB from July 2019 to June 2021 and who met the inclusion criteria were considered for the study.

### Study variables

**Sputum smear conversion after two months of TB treatment** was dichotomized into **converted and not converted** for patients with a negative follow up sputum smear results and for patients with a positive follow up sputum smear result respectively. As per the TB treatment guidelines in Rwanda, after two months of treatment, smear sputum is tested as a follow up exam, expecting conversion, a positive sign towards treatment response. This was done for all patients under TB patients enrolled in this study.

**Age** was categorized in four groups: <20 years, 20–39 years, 40–59 years, and 60 years and above.

#### Sex

is male or female.

#### Residence

was categorized in four provinces - Kigali city, Eastern, Western, Northern and Southern province.

#### HIV status

was dichotomized into positive or negative.

#### History of HIV

was categorized in three statuses - newly tested positive (those who knew their HIV positive at the time of TB diagnosis), people living with HIV (those who knew their HIV positive status even before TB diagnosis) and HIV negative for people who not having HIV.

#### On ART

was put into three treatment categories - Yes for patients taking ART, no for patients not taking ART and Not applicable for those not having HIV.

#### On cotrimoxazole

was categorized as - Yes for HIV positive patients receiving cotrimoxazole, No for HIV positive patients not taking cotrimoxazole and not applicable for HIV negative patients.

**BMI at the initiation of TB treatment** was divided into two group: BMI less than 18.5, and BMI equal and greater than18.5.

#### Previous treatment history

was categorized into patients who took TB treatments for the first time were categorized as new, those who had been diagnosed with TB for the second or several times were categorized as relapse, patients who had history of first-line treatment failure were categorized as treatment after failure of first-line treatment, and those who had history of TB infections but did not remember if they had failed their treatments were categorized as other previously treated, and finally those who had been lost while taking their treatment were categorized as treatment after lost to follow up.

#### Contact of index TB

dichotomized as Yes or No.

#### Contact of MDR-TB

dichotomized as Yes or No.

#### Diabetes

was categorized in three entities - Yes, No and unknown for those who did not know their diabetic status.

#### TB-nutrition support provided

was dichotomized as Yes or No.

#### Follow up by a community health worker (CHWs)

was dichotomized as Yes or No.

### Data collection and analysis methods

All patient’s information are collected at TB clinic in patient’s file during TB diagnosis, then it is entered in e-TB system by data managers. Data quality and accuracy is checked every quarter through data quality assessment meeting conducted by the central level at every district, where patients’ files are reviewed and cross-checked against the e-TB system records to detect any discrepancies in data accuracy and completeness. This process of data collection is done in all the district across the country because e-TB system is functional in every TB clinic both in urban and rural areas. During data collection of this study, SPPTB patients ‘records were extracted from e-TB in to Microsoft Excel and then cleaned by the principal investigator. The cleaned dataset was then exported to Stata version 16 for analysis (Stata Corp LLC). Sociodemographic and clinical characteristics of patients were described with frequencies and proportions. A bivariate logistic regression was computed between each independent variable and the dependent variable (Sputum smear conversion after two months of treatment) to determine if there was a significant association. Multivariable logistic regression analysis was then performed for all variables which were statistically significant in the bivariate logistic regression analysis to determine factors independently associated with the dependent variable. Adjusted odds ratio (AOR), confidence interval (CI), and p-value were considered statistically significant at 95% confidence level.

### Ethical considerations

The study was approved by the University of Rwanda, College of Medicine and Health Sciences, Institutional Review Board (IRB) with Reference number 280/CMHS IRB. In addition, a confidentiality agreement about handling patients’ information was made between the investigator and Rwanda Biomedical Center (RBC). During the data analysis, encryption was performed for patients’ identifiers like names and Tracnet numbers for HIV-positive patients. The full dataset was locked with a password known only by the principal investigator.

## Results

**Fig. 1:**
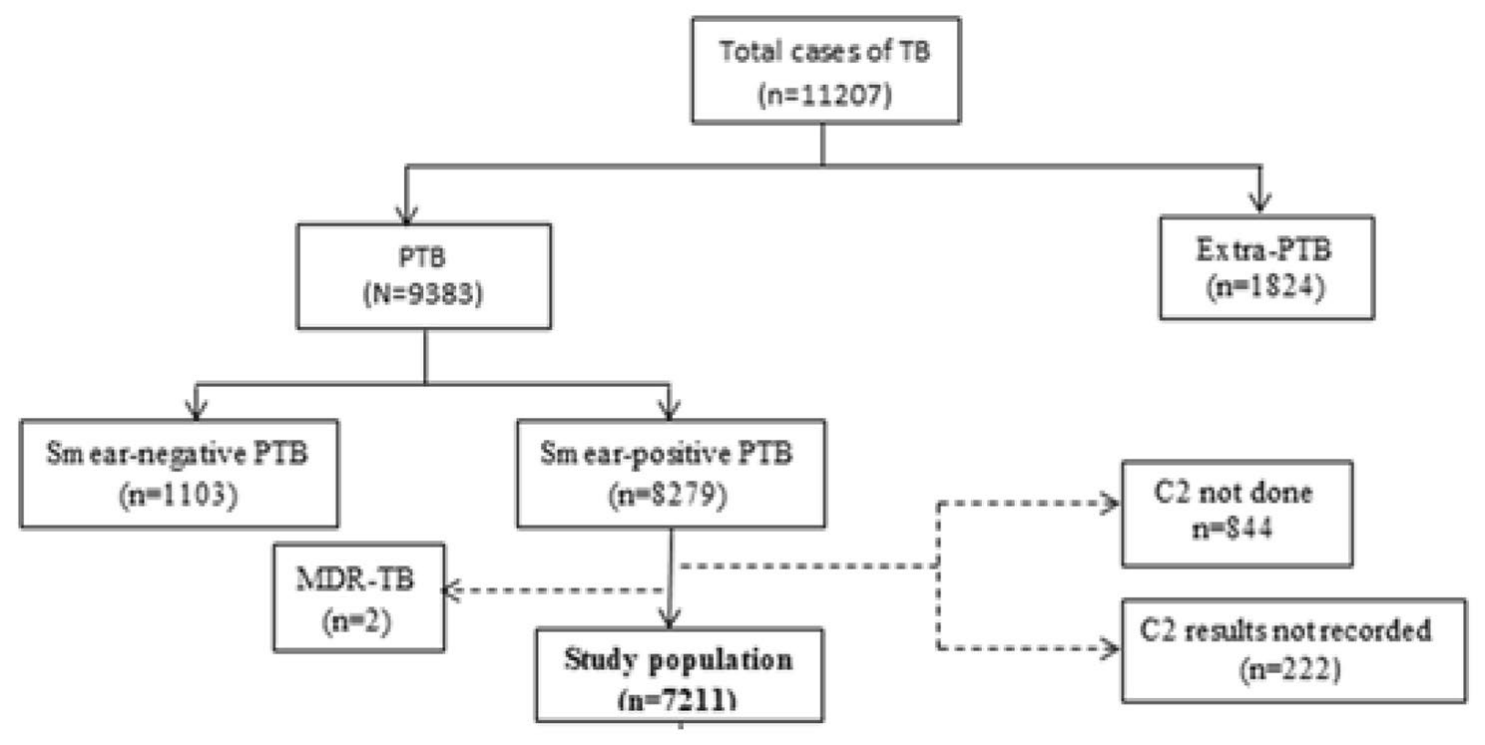
Algorithm for sampling of smear-positive pulmonary TB patients reported in Rwanda from July 2019 to June 2021

During the study period, 11,207 TB cases were reported in e-TB. Of this figure, 1824 (16.3%) were extra-pulmonary TB (EPTB) cases, and 1104 (9.8%) were smear-negative pulmonary TB (SNPTB). The EPTB and SNPTB cases were excluded from the analysis. Of the total 8279 (73.8%) SPPTB cases, follow-up sputum smear examination after the 2nd month of TB treatment was not done for 844 cases (10.2%) and two cases (0.02%) had Multidrug-Resistant Tuberculosis (MDR-TB), while 222 cases (2%) did not have documented follow-up sputum smear result after the second month of TB treatment, so they were also excluded from our study. The final cases that were included in this study were 7211 (87%) who had recorded a follow-up sputum smear result after the 2nd month of TB treatment (Fig. 1).

### Characteristics of the study participants

From July 2019 to June 2021, 7211 SPPTB cases were reported in e-TB countrywide. Of these around three quarters of the patients (73.2%) were males and 26.7% were females, while 53.31% of participants were aged between 20 and 39 years old, and 28.3% lived in Kigali city. Of the total participants 18.2% were living with HIV, 9.6% were previously treated with TB, and 63.5% PTB had a BMI < 18.5 at TB treatment initiation. The prevalence of sputum smear non-conversion among SPPTB was 9% and when assessing the proportion of non-converters by province, the Northern Province had the highest proportion of non-converters (12.2%). The prevalence of non-conversion was high (10%) among patient aged 40–59 years compared to other age groups (Tables 1 and 2).

**Table 1:** Socio-demographic characteristics of study participants (N = 7211)

Socio-demographic characteristics	Frequency (n)	Percentage (%)
**Age-groups**		
< 20years	322	4.47
20–39 years	3844	53.31
40–59 years	2245	31.13
60 years and above	800	11.09
**Gender**		
Male	5281	73.24
Female	1930	26.76
**Residence (Province)**		
East	1774	24.6
Kigali city	2041	28.3
North	657	9.11
South	1630	22.6
West	1109	15.38

**Table 2:** Clinical characteristics of study participants

Clinical characteristics	Frequency (n)	Percentage (%)
**HIV status**		
Negative	5897	81.78
Positive	1314	18.22
**History of HIV**		
HIV negative	5897	81.78
Newly tested Positive	362	5.02
People living with HIV	952	13.20
**TB treatment History**		
New	6521	90.43
Relapse	575	7.97
Treatment after failure of the first line	78	1.08
Treatment after lost to follow-up	27	0.37
Other previously treated	10	0.14
**Currently on ART**		
Yes	1239	17.18
No	75	1.04
Not applicable	5897	81.78
**Currently on cotrimoxazole**		
Yes	968	13.42
No	346	4.80
Not applicable	5897	81.78
**Diabetes**		
No	4559	63.22
Yes	41	0.57
Unknown	2611	36.21
**Contact with PTB +**		
No	6475	89.79
yes	736	10.21
**Contact with MDR-TB**		
No	7156	99.24
Yes	55	0.76
**Followed by CHWs**		
No	3473	48.16
Yes	3738	51.84
**BMI at treatment initiation**		
BMI < 18.5	4581	63.53
BMI > = 18.5	2630	36.47
**TB-nutrition support provided**		
Yes	1228	17.03
No	5983	82.97

The mean patient age included in the analysis was 39 ± 14.68 years with 5281(73.24%) being males and 1930 (26.76%) females.

### Analysis of the association between SPPTB patients’ characteristics and sputum smear non-conversion in Rwanda

During the bivariate logistic regression, factors like age, residence of the patient, history of first-line TB treatment failure, BMI, follow up by CHWs, were significantly associated with sputum smear non-conversion. Patients aged between 20 and 39 years old were 1.7 times more likely to have sputum smear non-conversion (OR = 1.7, 95% CI: 1.01–2.8), and those aged between 40 and 59 years old were twice more likely to have sputum smear non-conversion (OR = 2, 95% CI: 1.2–3.3) compared to patients less than 20 years old. Patients who lived in the Northern province were 1.4 times more likely to have sputum smear non-conversion after 2 months of treatment (OR = 1.4, 95% CI: 1.6-2) compared to those in the western province (Table 3).

**Table 3:** Association between socio-demographic characteristics and sputum smear non-conversion after 2 months of TB treatment among SPPTB patients in Rwanda, July 2019 to June 2021

Socio-demographic features	Two-months sputum smear outcome				
	Converted	Not converted			
	N (%)	N (%)	COR	95% CI	P-value
**Age category**					
< 20years	305 (94.7)	17 (5.3)	Ref		
20–39 years	3516 (91.5)	328 (8.5)	1.7	1.01–2.8	0.044
40–59 years	2021 (90)	224 (10)	2.0	1.2–3.3	0.008
60 years and above	737 (92.1)	63 (7.9)	1.5	0.9–2.7	0.129
**Sex**					
Male	4812 (91.1)	469 (8.8)	1.06	0.9–1.3	0.563
Female	1767 (91.5)	163 (8.5)	Ref		
**Residence (Province)**					
East	1613 (90.9)	161 (9.1)	1.04	0.8–1.3	0.763
Kigali city	1888 (92.5)	153 (7.5)	0.84	0.6–1.1	0.215
North	577 (87.8)	80 (12.2)	1.44	1.06–1.1	0.021
South	1489 (91.3)	141 (8.7)	0.9	0.75–1.3	0.93
West	1012 (91.3)	97 (8.7)	Ref		

Patients with a history of first-line TB treatment failure were 2.1 times more likely to have sputum smear non-conversion after two months of treatment (OR = 2.1, 95% CI: 1.2–3.9) compared to those who were treated for the first time (New). Patients with BMI less than 18.5 at TB treatment initiation were 1.5 times more likely to have sputum smear non-conversion after two months of treatment (OR = 1.5, 95% CI: 1.2–1.8) compared to those with BMI > = 18.5. Patients who were being followed up by CHWs during TB treatment were 1.3 times more likely to have sputum smear non-conversion after two months of treatment (OR = 1.3, 95% CI: 1.1–1.6) compared to those who were not followed up by CHWs (Table 4).

**Table 4:** Association between clinical characteristics and sputum smear non-conversion after 2 months of TB treatment among SPPTB patients in Rwanda, July 2019 to June 2021

Clinical features	Converted	Not converted			P-value
	N (%)	N (%)	COR	95% CI	
**HIV status**					
Negative	5363 (90.9)	534 (9.1)	Ref		
Positive	1216 (92.5)	98 (7.5)	0.8	0.64–1.01	0.064
**History of HIV**					
HIV negative	5363 (90.9)	534 (9.1)	Ref		
Newly tested Positive	332 (91.7)	30 (8.3)	0.9	0.6–1.3	0.62
People living with HIV	884 (92.9)	68 (7.1)	0.8	0.59-1.0	0.054
**TB treatment History**					
New	5958 (91.4)	563 (8.6)	Ref		
Relapse	524 (91.1)	51 (8.9)	1.03	0.8–1.4	0.847
Treatment after failure of the first-line	65 (83.3)	13 (16.7)	2.1	1.1–3.9	0.015
Treatment after lost to follow up	22 (81.5)	5 (18.5)	2.4	0.9–6.4	0.078
Other previously treated	10 (100)	0	1	Empty	
**Currently on ART**					
Yes	1147 (92.6)	92 (7.4)	Ref		
No	69 (92)	6 (8)	1.1	0.4–2.5	0.854
Not applicable	5363 (91)	534 (9)	1.2	0.9–1.5	0.066
**Currently on cotrimoxazole**					
Yes	897 (92.7)	71 (7.3)	Ref		
No	319 (92.2)	27 (7.8)	1.1	0.7–1.7	0.776
Not applicable	5363 (91)	534 (9)	1.2	0.9–1.6	0.081
**Contact with PTB +**					
No	5906 (91.2)	569 (8.8)	Ref		
yes	673 (91.4)	63 (8.6)	0.9	0.7–1.3	0.836
**Followed by CHWs**					
No	3211 (92.5)	262 (7.5)	Ref		
Yes	3368 (90.1)	370 (9.9)	1.3	1.1–1.6	< 0.001
**BMI at treatment initiation**					
BMI < 18.5	4130 (90.2)	451 (9.8)	1.5	1.2–1.8	< 0.001
BMI > = 18.5	2449 (93.1)	181 (6.9)	Ref		
**TB-nutrition support provided**					
Yes	1112 (90.5)	116 (9.5)	Ref		
No	5467 (91.4)	516 (8.6)	0.9	0.7–1.1	0.354

### Factors associated with sputum smear non-conversion among SPPTB patients

We conducted a multivariable logistic regression analysis for all variables that were statistically significant in the bivariate model. A number of factors were found to be statistically associated with sputum smear non-conversion after two months of treatment. Patients aged 20–39 years were 1.7 more likely to have sputum smear non-conversion (AOR = 1.7, 95% CI: 1.04–2.86) and those aged 40–59 years were twice more likely to have sputum smear non-conversion (AOR = 2, 95% CI: 1.18–3.3) compared to patients less than 20 years old. Patients living in the Northern Province were 1.4 times more likely to have sputum smear non-conversion (AOR = 1.4, 95% CI: 1.05-2.0) compared to those living in the Western Province. Patients with a history of first-line TB treatment failure were twice more likely to have sputum smear non-conversion (AOR = 2, 95% CI: 1.1–3.6) compared to those who were treated for the first time. Patients followed up by CHWs during TB treatment were 1.2 times more likely to have sputum smear non-conversion (AOR = 1.2, 95% CI: 1.04–1.5) compared to those who are followed-up in clinics and patients with BMI less than 18.5 at TB initiation were 1.5 times more likely to have sputum smear non-conversion (AOR = 1.5, 95% CI: 1.25–1.8) compared to those with BMI > = 18.5 (Table 5).

**Table 5:** **Factors associated with sputum smear no-conversion after two months of TB treatment among smear-positive PTB patients in Rwanda from July 2019 to June 2021**

Factors	Odds ratio	95% CI	P-value
Age category			
< 20 years	Ref		
20–39 years	1.7	1.04–2.8	0.033
40–59 years	2	1.18–3.3	0.009
60 years and above	1.4	0.81–2.5	0.207
**Residence (Province)**			
West	Ref		
East	0.9	0.75–1.3	0.914
Kigali city	0.9	0.69–1.2	0.543
North	1.4	1.05-2.0	0.022
South	0.9	0.71–1.2	0.642
**TB treatment history**			
New	Ref		
Relapse	1.01	0.75–1.4	0.905
Treatment after failure of first-line	2	1.1–3.6	0.028
Treatment after lost to follow up	2.4	0.9–6.5	0.073
Other previously treated	1	Empty	
**Followed by CHWs**			
No	Ref		
Yes	1.2	1.04–1.5	0.013
**BMI at treatment initiation**			
BMI less than 18.5	1.5	1.25–1.8	< 0.001
BMI > 18.5	Ref		

## Discussion

In this study, we explored the factors associated with sputum smear non-conversion after 2 months of treatment among SPPTB patients. Our analysis of 7211 PTB cases with documented follow-up sputum smear results after the second month of treatment, between July 2019 and June 2021, showed that the overall sputum smear non-conversion rate was 9%. This is transformed into a smear conversion rate of 91% which is significantly higher than the conversion rate of 78% and 82.8 revealed by studies conducted in Mumbai and Kota Kinabalu Malaysia respectively [17, 18]. We also found that age (20–39, 40-59years), residence (Northern Province), history of first-line treatment failure, being followed up by CHWs, and BMI less than 18.5 at TB treatment initiation were independently associated with sputum smear non-conversion after 2 months of treatment.

Surprisingly, in this study, we found that smear positive PTB patients aged between 20 and 39 years had a significantly high risk of having sputum smear non-conversion after 2 months of treatment. Our findings were different from the results shown by a study conducted in YAOUNDÉ, Cameroon which revealed that there was no significant association between age below 40 years and sputum smear non-conversion after 2 months of treatment [19]. This risk of having sputum smear non-conversion after 2 months of treatment among people in this age group of 20 to 39 years old could be due to a broadening of social contacts which is common among young adults and it is also exacerbated by the fact that most of the Rwandan population is young [20, 21]. The evidence suggests that current models of care are not meeting the needs of adolescent and young adults and this highlight issues of that need to be addressed and areas that require further study [20]. As expected, in this study, we have seen that the age groups of 40–59 years and 60 years and above were significantly associated with sputum smear non-conversion after 2 months of treatment. These findings are consistent with those of the study conducted in Sahary Hospital, Riyadh, Saudi Arabia where reduced immunity and late presentation for diagnosis among the elderly were highlighted as plausible reasons [22]. In our findings, SPPTB patients living in the Northern Province of Rwanda were 1.4 times more likely to have sputum smear non-conversion after 2 months of treatment compared to others living in the Western province. Surprisingly, the Northern Province was the one with a low proportion 657 (9.1%) of SPPTB cases countrywide. The association between Northern province and smear non-conversion could be due to the fact that apart from Kigali City, the Northern Province has the lowest number of TB clinics (99; 18.6%) compared to other upcountry provinces (Southern Province:142 (25.9%), Western Province:131 (23.9%), Eastern Province:129 (23.5%), and Kigali City: 47 (8.6%) TB clinics).

In most studies, people with HIV positive were more likely to have sputum smear non-conversion after 2 months of treatment compared to others without HIV [6, 12]. These findings were quite different from our study findings where we found that there was no association between HIV positive and sputum smear non-conversion after 2 months of treatment. This could be due to the specialist-driven treatment adherence training provided to HIV-positive patients that also had a positive impact on these patients while they were taking their anti-TB treatments, thereby influencing their two months sputum conversion. In line with research conducted in other settings [3, 6], patients with diabetes mellitus (DM) had a higher risk for two-month sputum smear non-conversion than those without DM. However, we could not evaluate DM as a risk factor since only forty-one patients had DM in our study. This number is relatively low, compared to that in the above-mentioned studies. In our study, SPPTB patients who had a history of first-line treatment failure (re-treatment) were twice more likely to have sputum smear non-conversion after 2 months of treatment compared to those who were new to anti-TB treatment. These findings were similar to those of studies conducted in Sharkia Governorate and northern Malaysia [21, 22]. Re-treated PTB patients found to have sputum smear non-conversion after 2 months of treatments would be examined additionally by sputum culture and be treated further based on drug sensitivity testing [23].

The present study reported a significant association between BMI less than 18.5 at TB treatment initiation and two-month sputum smear non-conversion. Similar findings were reported by several studies [6, 24, 25]. A possible explanation for this is that undernutrition tends to increase disease complications (low immunity), thereby reducing the chance of sputum smear conversion [26]. Moreover, PTB patients who were being followed up by CHWs during TB treatment were 1.2 times more likely to have sputum smear non-conversion after 2 months of treatment compared to those who were being followed up by clinicians. This could be a result of limited service provided by CHWs due to their heavy workload, and it may also be possibly due to different referral factors such as referring patients to CHWs with low treatment adherence level or facing other psychosocial problems that can affect their health status. Several studies conducted in different countries like Brazil [27], Ethiopia [33], Malawi [34], South Africa [35], and Uganda [36] showed that using CHWs in the follow up of non-hospitalized TB patients was a cost-effective alternative to the hospital-based treatment approach, but one study pointed out the importance of accurate training and supervision of CHWs in attaining universal health goals [36].

### Limitation of the study

The main limitation of our study was linked to the use of system data (secondary data). For some study subjects, there was a problem of missing data. Moreover, the reporting system did not capture some data of essential variables which might have an influence on sputum smear non-conversion of SPPTB patients, like clinical variables (adherence level, and viral load), socio-economic and socio-demographic characteristics (income, educational level, marital status, distance to the health facility) as well as behavioral factors (knowledge and attitude toward TB, alcohol consumption, smoking habit, illicit drug use) were not recorded. We also have to consider that excluding people who died or dropped out of care can influence determination of who fares poorly with TB treatment (do not convert their sputum). Some could have died because they couldn’t clear the organism because they were older, immunocompromised, malnourished, have HIV, so that you will have less patients with those variables alive or in care at 2 months post treatment. Also, HIV positive patients tend to have more smear negative TB disease and these are excluded at the initial from the study.

## Conclusion

Our study showed that sputum smear non-conversion at 9% is considered low and acceptable in following WHO targets and recommendations. We also found age (20–39, 40–59 years), residing in the Northern Province, and history of first-line treatment failure, being followed up by CHWs, and BMI less than 18.5 at TB treatment initiation to be independently associated with sputum smear non-conversion after 2 months of treatment. Operational research is needed to explore innovative approach to deal with sputum smear non-conversion after two months of TB treatment among SPPTB in Rwanda especially in the northern province and in patients followed up by CHWs.

## Data Availability

The datasets analyzed during the current study are available from the corresponding author on reasonable request.

## List of Abbreviations

AFB: Acid-Fast Bacilli
AOR: Adjusted Odds Ratio
ART: Antiretroviral Therapy
BC: Bacteriologically Confirmed
BMI: Body Mass Index
CHWs: Community Health Workers
CI: Confidence Interval
COR: Crude Odds Ratio
DM: Diabetes Mellitus
EPTB: Extra-Pulmonary Tuberculosis
FETP: Field Epidemiology and Training Program
HIV: Human Immunodeficiency Virus
FY: Fiscal Year
IRB: Institutional Review Board
MDR: Multi-Drug Resistant
MTB: Mycobacterium Tuberculosis
PTB: Pulmonary Tuberculosis
SNPTB: Smear Negative Pulmonary Tuberculosis
SPPTB: Smear Positive Pulmonary Tuberculosis
TB: Tuberculosis
WHO: World Health Organization

## Declarations

We declare that this research entitled factors associated with sputum smear non-conversion after two months of tuberculosis treatment among smear-positive pulmonary tuberculosis patients in Rwanda is original research. It has not been submitted anywhere for publication purpose. The collaborative and cited contributions have been indicated clearly and acknowledged where references have been provided on all supporting literatures and resources.
